# The National Dental Association

**Published:** 1879-06

**Authors:** 


### EDITORIAL, ETC.

*The National Dental Association.*—This Association, late "Southern," as will be seen by the notice in the May and June Nos. of the *Journal,* will hold its eleventh annual session at Augusta, Georgia, commencing July 8th, 1879.

A large number of the most prominent members of the dental profession are exerting their utmost efforts to further the interests of this Association, to make it truly national in its character and objects, and to place it upon a firm and permanent basis.

The officers elected at the Niagara meeting of 1878, are actively at work, and the most cheering reports are received from all quarters, giving the best indication that this Augusta meeting will be the largest dental assembly ever held in the southern part of this country. No less than three State Societies will be present, those of Georgia, North and South Carolina, and there is an interest manifested in different sections of the country, North as well as South, which promises a full representation.

We urge upon all who have the interests of their profession at heart to make every effort to attend this Augusta meeting, where, from the character, and professional standing of those who will be present, much that is useful may be learned, and the objects of a National Association clearly demonstrated. All dental depots are invited to exhibit their manufactures, and from the replies already received there is not a doubt, but that the display of all the recently invented and improved dental instruments, appliances, materials, etc., etc., will be one of the largest ever made on an occasion of the kind.

Dr. L. D. Carpenter, of Atlanta, Ga., the 1st Vice President of the Association, on account of the illness of Dr. A. C. Ford, the Corresponding Secretary, volunteered to assume the duties of this position, and has been actively at work for months past in doing all that is possible to make the Augusta meeting a grand success. The Recording Secretary, Dr. E. S. Chisholm, of Tuscaloosa, Ala., is also doing all that such a position requires, in the same direction. The Executive Committee, of which Dr. W. C. Wardlaw, of Augusta, Ga., is Chairman, has been actively engaged in making all the necessary arrangements for the meeting, and from all of these officers the most favorable accounts have been received.

The Chairmen and members of the different standing committees are responding nobly, and all interested in this *National* Association are encouraged and hopeful.

Dental practitioners from every part of the country are cordially invited, and will be kindly welcomed.

This meeting being held at a season of the year when the dentist can best afford to be absent from his practice, and also when recreation is beneficial to health, and a change from the daily routine of office life not only desirable but necessary, all who can avail themselves of this opportunity should do so, and thus perform a duty they owe to their profession as well as to themselves.

